# Virtual reality-based and eye tracking-assisted attention refocusing training for adult Attention-Deficit/Hyperactivity Disorder

**DOI:** 10.1192/j.eurpsy.2022.2251

**Published:** 2022-09-01

**Authors:** B. Selaskowski, L. Asché, A. Wiebe, K. Kannen, B. Aslan, D. Sanchéz, S. Lux, A. Philipsen, N. Braun

**Affiliations:** University Hospital Bonn, Department Of Psychiatry And Psychotherapy, Bonn, Germany

**Keywords:** attention-deficit/hyperactivity disorder, virtual reality, adults

## Abstract

**Introduction:**

Neurofeedback regimes in the treatment of adult ADHD are commonly EEG-based and have several shortcomings, including a weak signal-to-noise ratio, low transfer rates from laboratory to everyday environments and ambiguous evidence in respect to adequate brain signals of interest.

**Objectives:**

To investigate, if an eyetracking-based real-time feedback in a virtual environment can enhance attentional performance, as measured by behavioral, EEG and eyetracking parameters.

**Methods:**

Overall, n=18 adult patients with ADHD and n=18 healthy controls (HC) performed a continuous performance task (CPT) in a virtual seminar room, while distracting virtual events occurred. In case the participant’s gaze drifted away from the task an automated audiovisual feedback indicated the participant to refocus on the task. Three 20-minutes blocks were presented in counter-balanced order, that differed in respect to whether real feedback, sham feedback or no feedback was additionally provided.

**Results:**

Mixed ANOVAs with within-subject factors ‘Condition’ (real feedback, sham feedback, no feedback) and ‘Phase’ (distractor phases vs. non-distractor phases) and a between-factor ‘Group’ (ADHD patients vs. HC) revealed better task performances in HC than ADHD patients in respect to omission errors (*p* = .023), mean reaction times (*p* = .042) and reaction time variabilities (*p* = .007; cf. Figure 1). Moreover, omission errors turned to be higher during distractor-present than distractor-absent trials (*p* = .007), especially in ADHD.

**Figure 1.**
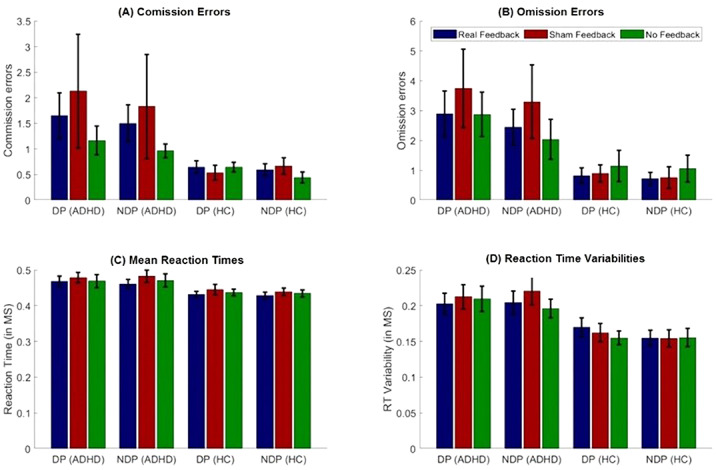
CPT results. DP=distractor-phases, NDP=non-distractor-phases

**Conclusions:**

While the virtual CPT turns out to discriminate well between patients with ADHD and HC, the behavioral results do not indicate an attentional performance enhancement based on the gaze-dependent feedback.

**Disclosure:**

No significant relationships.

